# The effects of flipped learning for bystander cardiopulmonary resuscitation on undergraduate medical students

**DOI:** 10.5116/ijme.5a2b.ae56

**Published:** 2017-12-22

**Authors:** Taizo Nakanishi, Tadahiro Goto, Taketsune Kobuchi, Tetsuya Kimura, Hiroyuki Hayashi, Yasuharu Tokuda

**Affiliations:** 1 Department of Emergency Medicine, Fukui Prefectural Hospital, Fukui, Japan; 2 Department of Emergency Medicine, Massachusetts General Hospital, Boston, USA; 3 Department of Emergency Medicine, University of Fukui Hospital, Fukui, Japan; 4 Muribushi Okinawa for Teaching Hospitals, Okinawa, Japan

**Keywords:** Cardiopulmonary resuscitation, Flipped learning

## Abstract

**Objectives:**

To compare bystander cardiopulmonary resuscitation skills retention between conventional learning and flipped learning for first-year medical students.

**Methods:**

A post-test only control group design. A total of 108 participants were randomly assigned to either the conventional learning or flipped learning. The primary outcome measures of time to the first chest compression and the number of total chest compressions during a 2-minute test period 6 month after the training were assessed with the Mann-Whitney U test.

**Results:**

Fifty participants (92.6%) in the conventional learning group and 45 participants (83.3%) in the flipped learning group completed the study. There were no statistically significant differences 6 months after the training in the time to the first chest compression of 33.0 seconds (interquartile range, 24.0-42.0) for the conventional learning group and 31.0 seconds (interquartile range, 25.0-41.0) for the flipped learning group (U=1171.0, p=0.73) or in the number of total chest compressions of 101.5 (interquartile range, 90.8-124.0) for the conventional learning group and 104.0 (interquartile range, 91.0-121.0) for the flipped learning group (U=1083.0, p=0.75). The 95% confidence interval of the difference between means of the number of total chest compressions 6 months after the training did not exceed a clinically important difference defined a priori.

**Conclusions:**

There were no significant differences between the conventional learning group and the flipped learning group in our main outcomes. Flipped learning might be comparable to conventional learning, and seems a promising approach which requires fewer resources and enables student-centered learning without compromising the acquisition of CPR skills.

## Introduction

Sudden cardiac death (SCD) is a major public health problem, and it has been estimated that over 20,000 SCD cases occur annually in Japan.^1^ Several community-based studies have reported that the incidence of SCD in Japan was stable for decades.^2^^-^^4^ However, there is a concern that the incidence of SCD might increase in the near future because of the growing number of patients who have ischemic heart disease or its risk factors, such as diabetes mellitus.^2^^,^^5^^,^^6^ Since successful treatment of SCD depends on the immediate performance of cardiovascular resuscitation, it is important to implement educational efforts for promoting good cardiovascular resuscitation skills.

Bystander cardiopulmonary resuscitation (CPR) plays a major role in increasing the survival rate from out-of-hospital cardiac arrest (OHCA), and it can double or triple the chance of surviving ventricular fibrillation.^7^ The rates of bystander CPR for OHCA have been improving but still remain low at 44.3% in Japan in 2012.^8^

Conventional learning in basic life support (BLS) consists of didactic and hands-on components and requires a class-based educational session that is usually time-consuming and expensive. Thus, various new approaches to provide BLS training have been developed to overcome these problems.^9^^-^^11^ However, there is currently limited data about the ideal training method for BLS. Although similar concepts were proposed more than a decade ago, some educators recently developed a novel educational model called flipped learning or a flipped classroom, also known as a backwards, inverted or reverse classroom.^12^^,^^13^ The flipped learning is a pedagogical approach in which the instructor-led lecture is done prior to class, while homework or assigned problems are done in the scheduled class.^14^ In typical flipped learning, learners watch short videos pre-recorded by instructors and study by themselves in preparation for a class. The content can be viewed by the learners as many times as necessary to master the knowledge at any time and at their own pace. This approach

allows for student-centered learning activities; that is, students can practice or deepen what they have learned by discussing with the teachers and their classmates in the class. Through this process, students can master their problem-solving skills and have more hands-on training during the dedicated class times.^15^^, ^^16^

We aimed to evaluate and compare CPR skills retention between conventional and flipped learning groups immediately and 6 months after the training. We hypothesized that the rescuers who have learned bystander CPR using the flipped learning method would demonstrate better CPR performance than those who have learned it through the conventional learning.

## Methods

### Study design and setting

This study was a post-test only control group design to evaluate the effectiveness of flipped learning compared to conventional learning for CPR.^17^ The study was conducted at the medical faculty of the University of Fukui, Japan.

### Sampling and recruitment

Study participants were recruited from the 110 registered first-year medical students at the medical faculty of the University of Fukui, Japan. All students have agreed to participate in the study. The students knew this assessment of CPR performance would not be considered in their grades.

The 110 students in the first-year medical school class were then randomly assigned to either the conventional learning group or the flipped learning group. Both groups had one participant who did not attend. Thus, 108 participants were included in this study. Thirteen participants (12.0%) did not attend the 6-month follow-up evaluation. Overall, 50 students in the conventional learning group (92.6%) and 45 students in the flipped learning group (83.3%) have completed the study.

There were no significant differences in the demographic characteristics including age, gender, any experience of recent CPR training (defined as CPR training in the previous 2 years) and actual CPR, their own or family's history of heart disease and family history of sudden cardiac death between the two groups (Table 1). Two participants in the flipped learning group (3.7%) reported that they did not watch the video at all. Nevertheless, they were included in this study. Thirteen participants who did not complete the study were not significantly different in the demographic characteristics from the participants who completed it in both groups.

The risk of harmful events for the participants was low since our study was a simulation-based study. Consent was obtained from all of the research participants, and it was explained that their participation in this study was completely voluntary. The study protocol was approved by the Ethics Committee of the University of Fukui Hospital.

**Table 1:** Demographic characteristics (N=108)

Characteristic	Conventional learning n=54	Flipped learning n=54	p-value
Age (Mean ± SD)	19.4 ± 1.6	19.3 ± 2.0	0.76
	n (%)	n (%)	
Male	34 (61.8)	34 (61.8)	0.99
Recent CPR training^*^	34 (61.8)	36 (65.5)	0.84
Experience of actual CPR	0	0	0.99
Their own or family's history of heart disease	2 (3.6)	1 (1.8)	0.99
Family history of sudden cardiac death	2 (3.6)	0	0.50
Number of times to watch the video			
0		2 (3.7)	
1		47 (87.0)	
2		4 (7.4)	
3		1 (1.8)	

CPR = cardiopulmonary resuscitation; SD = standard deviation *Recent CPR training is defined as CPR training in the previous 2 years

### Sample size

The sample size was calculated based on the number of chest compressions. It is known that a chest compression rate of 100 to 120 per minute is optimal.^18^^,^^19^ In this study, the 2-minute test period included the initial assessment in order to simulate a real setting. We estimated that the actual time for chest compressions would be 1 minute, eliminating the time for the initial assessment and mouth-to-mouth ventilation from previous studies.^20^^,^^21^ Therefore, we assumed that the clinically important difference in the number of total chest compressions was 20. Under the condition of an alpha error of 5% and a power of 80%, 36 participants per group were needed. Expecting a 20% dropout rate, the necessary sample size was estimated to be 90 participants in total.

### Data collection procedures

The participants' skills were assessed immediately after the conventional and flipped learning and 6 months later. Each participant was evaluated individually for 2 minutes according to a standardized testing protocol including an initial assessment, chest compressions and mouth-to-mouth ventilation by examiners who were blinded to the group assignment. All of the examiners were certified, and trained instructors of the ICLS course, and the authors were not involved in the evaluation.

**Table 2:** Participants’ performance of resuscitation immediately after training

Resuscitation skills	Conventional learning^*^n = 54	Flipped learning^*^n = 54	U-statistic	p-value
Time to first chest compression, second	29.5 (24.0-41.0)	34.0 (29.5-45.0)	1864.0	0.01^**^
Total chest compressions, n	120.0 (92.8-133.8)	101.0 (90.0-120.0)	998.5	0.005^**^
Hands-off time, second	33.0 (25.8-38.3)	34.0 (31.8-37.0)	1581.0	0.45
Correct hand positions, %	100.0 (62.8-100.0)	100.0 (69.5-100.0)	1481.5	0.87
Average chest compression depth, mm	48.50 (42.0-62.0)	53.0 (41.0-61.0)	1494.5	0.82
Appropriate chest recoil, %	69.0 (7.0-98.3)	79.5 (22.3-100.0)	1630.0	0.29
Appropriate chest compression depth, %	45.0 (7.0-99.0)	82.0 (2.0-97.3)	1493.5	0.83
Chest compression rate per minute, n	129.5 (117.0-142.0)	131.5 (120.0-141.3)	1526.5	0.67
Total ventilation, n	6.0 (3.8-7.0)	5.0 (3.0-6.0)	1280.0	0.27
Average ventilation volume, ml	481.5 (314.0-819.3)	645.5 (304.0-884.8)	1521.5	0.70
Appropriate ventilation volume, %	14.0 (0.0-40.8)	17.0 (0.0-50.0)	1553.0	0.54
Ventilation rate per minute, n	3.0 (1.8-4.0)	2.0 (1.0-3.0)	1193.5	0.10
Time from AED arrival to shock, second	55.0 (45.0-68.0)	60.0 (53.0-72.8)	1725.5	0.10
Key elements of CPR				
Safe approach	47 (87.0)	53 (98.2)		0.06
Check responsiveness	53 (98.2)	53 (98.2)		0.99
Call 119 (emergency phone number in Japan)	45 (83.3)	53 (98.2)		0.02^**^
Ask for automatic external defibrillator (AED)	54 (100)	54 (100)		0.99
Check breathing	48 (88.9)	49 (90.7)		0.99
Compression: ventilation = 30:2	49 (90.7)	49 (90.7)		0.99
Appropriate ventilation	49 (90.7)	50 (92.6)		0.99
Correct positioning of deﬁbrillator pads	52 (96.3)	52 (96.3)		0.99
Safe delivery of shock	42 (77.8)	41 (75.9)		0.99

*The figures inside the brackets are interquartile range **Statistically significant difference

**Table 3.** Participants’ performance of resuscitation 6 months after training

Resuscitation skills	Conventional learning^*^n = 50	Flipped learning^*^n = 45	U-statistic	p-value
Time to first chest compression, second	33.0 (24.0-42.0)	31.0 (25.0-41.0)	1171.0	0.73
Total chest compressions, n	101.5 (90.8-124.0)	104.0 (91.0-121.0)	1083.0	0.75
Hands-off time, second	34.5 (27.0-38.0)	34.0 (29.5-38.5)	1171.5	0.73
Correct hand positions, %	100.0 (98.8-100.0)	100.0 (72.5-100.0)	995.0	0.25
Average chest compression depth, mm	51.50 (43.5-57.0)	50.0 (38.5-58.5)	1018.5	0.43
Appropriate chest recoil, %	90.0 (53.8-100.0)	80.0 (32.0-99.5)	964.0	0.23
Appropriate chest compression depth, %	71.5 (9.8-97.3)	52.0 (0.0-98.0)	1053.0	0.59
Chest compression rate per minute, n	120.0 (111.0-132.5)	124.0 (118.0-131.5)	1329.0	0.13
Total ventilation, n	4.0 (2.0-6.0)	3.0 (0.0-6.0)	951.0	0.19
Average ventilation volume, ml	491.0 (279.5-777.8)	329.0 (0.0-509.5)	770.0	0.008^**^
Appropriate ventilation volume, %	12.5 (0.0-50.0)	0.0 (0.0-65.0)	1175.0	0.69
Ventilation rate per minute, n	2.0 (1.0-3.0)	1.0 (0.0-3.0)	968.0	0.23
Time from AED arrival to shock, second	57.5 (50.0-63.0)	57.0 (51.0-65.5)	1148.0	0.86
Key elements of CPR				
Safe approach	37 (74.0)	36 (80.0)		0.63
Check responsiveness	49 (98.0)	44 (97.8)		0.99
Call 119 (emergency phone number in Japan)	28 (56.0)	26 (57.8)		0.99
Ask for automatic external defibrillator (AED)	49 (98.0)	44 (97.8)		0.99
Check breathing	42 (84.0)	37 (82.2)		0.99
Compression: ventilation = 30:2	47 (94.0)	41 (91.1)		0.70
Appropriate ventilation	48 (96.0)	45 (100.0)		0.50
Correct positioning of deﬁbrillator pads	50 (100.0)	42 (93.3)		0.99
Safe delivery of shock	31 (62.0)	29 (64.4)		0.83

*The figures inside the brackets are interquartile range **Statistically significant difference

After the 2 minutes of CPR evaluation, the AED was brought to the participant, and their AED operation skills were evaluated. All CPR assessments were completed on the Resusci Anne mannequins connected to a SimPad SkillReporter. Before the first evaluation of CPR performance, each of the participants was asked to complete a questionnaire that included their age, sex, recent CPR training, any experience of actual CPR, their own or family's history of heart disease and their family's history of sudden cardiac death. The participants in the flipped learning group were also asked how many times they had watched the video.

Our primary outcome measures were the time to first chest compression and the number of total chest compressions performed during the 2-minute test period 6 months after training. The time to the first chest compression was defined as time from the start of CPR evaluation till the first chest compression performed in the evaluation. These were recorded by the Resusci Anne mannequins and the SimPad SkillReporter.

**Table 4:** Measure of total chest compressions 6 months after training

Measure	Conventional learning Mean (SD)	Flipped Learning Mean (SD)	95% CI	t-statistic	p-value
	n = 50	n = 45			
Total chest compressions	107.0 ± 24.2	104.5 ± 25.4	-7.6 - 12.6	0.49	0.62

Key: CI = confidence interval; SD= Standard Deviation

The secondary outcome measures were the time to the first chest compression and the number of total chest compressions performed during the 2-minute test period immediately after the training. Both immediately and 6 months after the training session, we evaluated the other outcome measures, including the hands-off time, percentage of correct hand positions, chest compression rate, average chest compression depth, percentage of appropriate chest recoil, percentage of appropriate chest compression depth, the number of total ventilation, average ventilation volume, percentage of appropriate ventilation volume and ventilation rate. These outcome measures were all recorded by the Resusci Anne mannequins and the SimPad SkillReporter. We also evaluated the time from AED arrival to shock and the key elements of CPR, which included the safe approach, check responsiveness, calling 119 (the emergency phone number in Japan), asking for an AED, breathing check, appropriate ratio of compression and ventilation, appropriate ventilation, correct positioning of deﬁbrillator pads and safe delivery of shock. These were also measured both immediately and 6 months after the training as other outcome measures. The time from AED arrival to shock was measured by the examiners, who gave either an 'adequate' or 'inadequate' grade for the key elements. The appropriate chest compression depth was defined as more than 50 mm, and the appropriate chest compression rate was defined as 100-120 per minute based on the 2010 American Heart Association (AHA) Guidelines.^22^ Hands-off time was defined as periods without compressions during resuscitation, and the appropriate ventilation volume was defined as 400-700 ml.

### Intervention

The participants were randomly assigned to either the 60-minute conventional learning group or the 30-minute flipped learning group. The randomization scheme was generated by using the web site.^23^

The conventional learning group participated in a 60-minute training program consisting of an initial assessment, chest compressions, mouth-to-mouth ventilation, and an automated external defibrillator (AED) operation based on the 2010 AHA Guidelines.^22^ After the instruction and discussion of CPR for 30 minutes, the participants received hands-on training similarly for 30 minutes. After the training session, all of the participants in the conventional learning group directly proceeded to their testing stations and their performance was assessed by the examiners.

We defined flipped learning in our study as the use of video learning followed by hands-on training. Specifically, the flipped learning group participants were directed to watch an official video provided by the Japanese Red Cross Society on the internet one week before the hands-on training. The video is approximately 15 minutes in length and explains how to perform bystander CPR based on the 2010 AHA Guidelines.^22^ The participants were instructed not to use other videos or resources in order to learn bystander CPR. On the day of the hands-on training, the flipped learning group participated in a 30-minute training program. The participants and instructors first discussed the video content, and all of the participants' questions were answered by the instructors before the training session started. We assumed that they had learned how to perform CPR from the video; therefore, no lecture was provided by the instructors. Thus, the training program in this group was 30 minutes shorter than the conventional group. They also proceeded to their testing stations immediately after the training.

Six months after the training sessions, all of the participants were invited to a reassessment, and their skills were evaluated by the examiners. This study was conducted as a part of their curriculum, so the participants were notified of the reassessment in advance. These training programs were conducted using Little Anne mannequins and the AED Trainer 2. Each group consisting of five participants was provided one mannequin and the AED trainer respectively. All participants were instructed by the physicians and nurses who were certified instructors of the Immediate Cardiac Life Support (ICLS) course or who had completed the ICLS course and had instruction experience equivalent to the certified instructors. The instructor/participant ratio was 1:5 in the both groups. None of the authors were involved in the instruction. The ICLS course is the most widely conducted course for CPR training in Japan and is managed by the Japanese Association for Acute Medicine.^24^

### Statistical analysis

Summary statistics were presented as the means with standard deviations or medians with interquartile ranges as appropriate. As most variables were not normally distributed, continuous variables were described using medians with interquartile ranges, and the Mann-Whitney U test was used to assess significant differences. In terms of our primary and secondary outcomes, we also performed Student's t-test if the distribution was normal. Fisher's exact test was used for binary variables. P-values < 0.05 were considered statistically significant. Statistical analyses were performed using SPSS Ver. 22 (SPSS, Inc., Chicago, IL, USA).

## Results

Table 2 displays the participants' performance immediately after the training and Table 3 displays the participants' performance 6 months after the training. There were no statistically significant differences 6 months after training between the two groups in our primary outcome measures, time to the first chest compression (33.0 [24.0-42.0] vs. 31.0 [25.0-41.0], U=1171.0, p=0.73) and the number of total chest compressions performed during the 2-minute test period (101.5 [90.8-124.0] vs. 104.0 [91.0-121.0], U=1083.0, p=0.75). The distribution of these primary outcome measures was normal, so we performed Student's t-test to calculate the 95% confidence interval (CI) of the difference between the means of the number of total chest compressions performed by the two groups 6 months after the training (Table 4). The 95% CI extended from -7.6 to 12.6, and it did not exceed 20, the number that we defined as a clinically important difference.

In terms of our secondary outcome measures, the time to the first chest compression immediately after training was significantly shorter in the conventional learning group than in the flipped learning group (29.5 vs 34.0, U=1864.0, p=0.01). Another secondary outcome, the number of total chest compressions performed during the 2-minute test period immediately after training, was significantly greater in the conventional learning group than in the flipped learning group (120.0 vs 101.0, U=998.5, p=0.005).

In other outcome measures that we have evaluated, the percentage of participants who did 'call 119' immediately after the training was 83.3% in the conventional learning group and 98.2% in the flipped learning group (p=0.02). The only significant difference found at the 6-month follow-up evaluation was in the average ventilation volume. The average ventilation volume was greater in the conventional learning group than in the flipped learning group (U=770.0, p=0.008). There were no significant differences in the remainder of other outcome measures.

## Discussion

In this simulation-based study, there were no significant differences in the means of total chest compressions between the conventional learning group and the flipped learning group 6 months after the training. Therefore, this study might indicate that conventional learning and flipped learning lead to comparable levels of retention of CPR skills.

Our results are consistent with those of other studies that investigated the effectiveness of video-based training for CPR.^9^^,^^25^^,^^26^ Although the video-based training was shorter in duration than the conventional training in some studies, including ours, there were no significant differences for the main learning outcomes between the conventional and video-based training groups in these previous studies.

There were significant differences in our secondary outcome measures between the two groups, and the conventional learning might be better than the flipped learning immediately after training. This could be explained by the longer instruction time in the conventional learning group. However, there were no significant differences in other outcome measures that suggested appropriate chest compressions including hands-off time, average chest compression depth and appropriate chest recoil.

In our other outcome measures, there was a significant difference in the percentage of participants who did 'call 119' between the two groups immediately after training. Nonetheless, the difference was not found 6 months after training. We noted that the importance of calling promptly was highlighted in the video and we assume this could cause the difference immediately after training.

We defined flipped learning in our study as a combination of learning using a 15-minute video followed by 30-minute hands-on training. Thus, instructors for the flipped learning group were needed for only half the time compared to the conventional learning group. We believe that flipped learning is a powerful and promising approach in adult learning, including medical education applications, and it may be especially useful in settings with fewer human resources.^16^^,^^27^ With flipped learning, students can learn from a video instead of receiving a lecture from a CPR instructor. Additionally, they can learn at the time and place of their choice and at their own pace, as they can rewind the video as needed.^12^^,^^15^^,^^16^^,^^28^

When introducing flipped learning, we did not need to make the videos because a large number of videos explaining how to perform bystander CPR are already available to the public. Flipped learning does not require new equipment or mannequins, and it is easy to adopt if learners have access to the internet.^12^^,^^13^

We aimed to show that the video learning was better than the conventional learning in the retention of CPR training in this study. Though, the significant difference was not detected between the conventional learning group and the video learning group six months after training. However, the training program we tested requires fewer resources and offers time efficiency and comfort for learners without compromising their acquisition of CPR skills. If using flipped learning could lead to comparable CPR skills retention compared to conventional learning in the training of laypersons, it would efficiently increase a number of rescuers able to perform CPR correctly.

### Limitations

There were several limitations to this study. First, this study was conducted as a part of the participants' medical school curriculum, so the participants were notified of the study process in advance, although they were randomly assigned to either group. Second, we used a video already uploaded to the internet and participants did not need to log in to view it. Thus, it was unclear how much or how deeply they learned from the video content. We asked the participants how many times they watched the video and only 2 participants (3.7%) reported that they did not watch it at all. We could overcome this concern by utilizing a log-in system or embedding a quiz or a brief test in the video.^16^^,^^28^ Third, our choice of participants might have influenced the outcomes of the study. They might be highly motivated because they were studying to become doctors, although they knew the results of CPR performance would not be considered in their grades. Fourth, it is important to note that this is a mannequin study. The mannequins we used are unable to represent humans, so appropriate CPR performance on a mannequin might not necessarily assure appropriate practice of CPR. Finally, our study was first intended to determine if flipped learning was superior to conventional learning. This study was neither a non-inferiority trial nor equivalence trial, so future research, preferably through a non-inferiority trial with more samples, would be needed to prove the non-inferiority of flipped learning over conventional learning in CPR education.

## Conclusions

There were no significant differences between the conventional learning group and the flipped learning group in the retention of CPR skills 6 months after training in our study. Thus, we could not demonstrate our research hypothesis that flipped learning was superior to conventional learning. However, flipped learning might be comparable to conventional learning due to the small difference. This novel approach would require fewer resources and offer greater time efficiency and comfort for learners without compromising their acquisition of CPR skills. Therefore, it could be a promising approach in order to propagate the CPR skills widely and efficiently. The future research would be needed to prove the non-inferiority of flipped learning over conventional learning in CPR training before its implementation. Further, it is also essential to confirm that the flipped learning is efficacious in the CPR skills retention for lay people in the future studies.

### Acknowledgments

We thank the Japanese Red Cross Society for permission to use their video for CPR training.

### Conflict of Interest

The authors declare that they have no conflict of interest.
